# ^1^H, ^13^C and ^15^N chemical shift assignment for stem-loop 5a from the 5‘UTR of HCoV-229E

**DOI:** 10.1007/s12104-025-10243-4

**Published:** 2025-07-31

**Authors:** Nina M. Krause, Anna Wacker, Christian Richter, Boris Fürtig, Ramakanth Madhugiri, John Ziebuhr, Harald Schwalbe

**Affiliations:** 1 Institute for Organic Chemistry and Chemical Biology, Johann Wolfgang Goethe University, Max-von-Laue-Straße7, 60438 Frankfurt/M, Germany; 2 Center for Biomolecular Magnetic Resonance (BMRZ), Johann Wolfgang Goethe University, Max-von-Laue‑Str. 9, 60438 Frankfurt/M, Germany; 3 Institute of Medical Virology, Justus Liebig University, Giessen, Germany

**Keywords:** Coronaviruses, HCoV-229E, 5‘-UTR, SL5a, Solution NMR spectroscopy

## Abstract

Due to the emergence of the SARS-CoV-2 virus, research on coronaviruses has been massively accelerated. In addition to SARS-CoV-2, there are other human coronaviruses, including HCoV-229E. In all coronaviruses, secondary structure predictions indicate the presence of conserved structural elements in the 5’-untranslated region (5’-UTR). These conserved elements play crucial roles in RNA translation and replication. Stem-loop 5 (SL5), consisting of three substructures (5a, 5b, 5c), is highly conserved and harbours the start codon for translation. SL5 has repetitive structural motifs (RSMs), 5’-UUYYGU-3’, which are conserved in many alpha- and betacoronaviruses. In the following, we present the ^1^H, ^13^C and ^15^N NMR resonance assignment of the SL5a RNA element from HCoV-229E and variations in the RSMs to show the effect of loop mutations on the structure of the hexaloop, revealing the different impact of each loop nucleotide on RNA dynamics.

**Supplementary Information:**

The online version contains supplementary material available at 10.1007/s12104-025-10243-4.

## Biological context

**Fig. 1:**
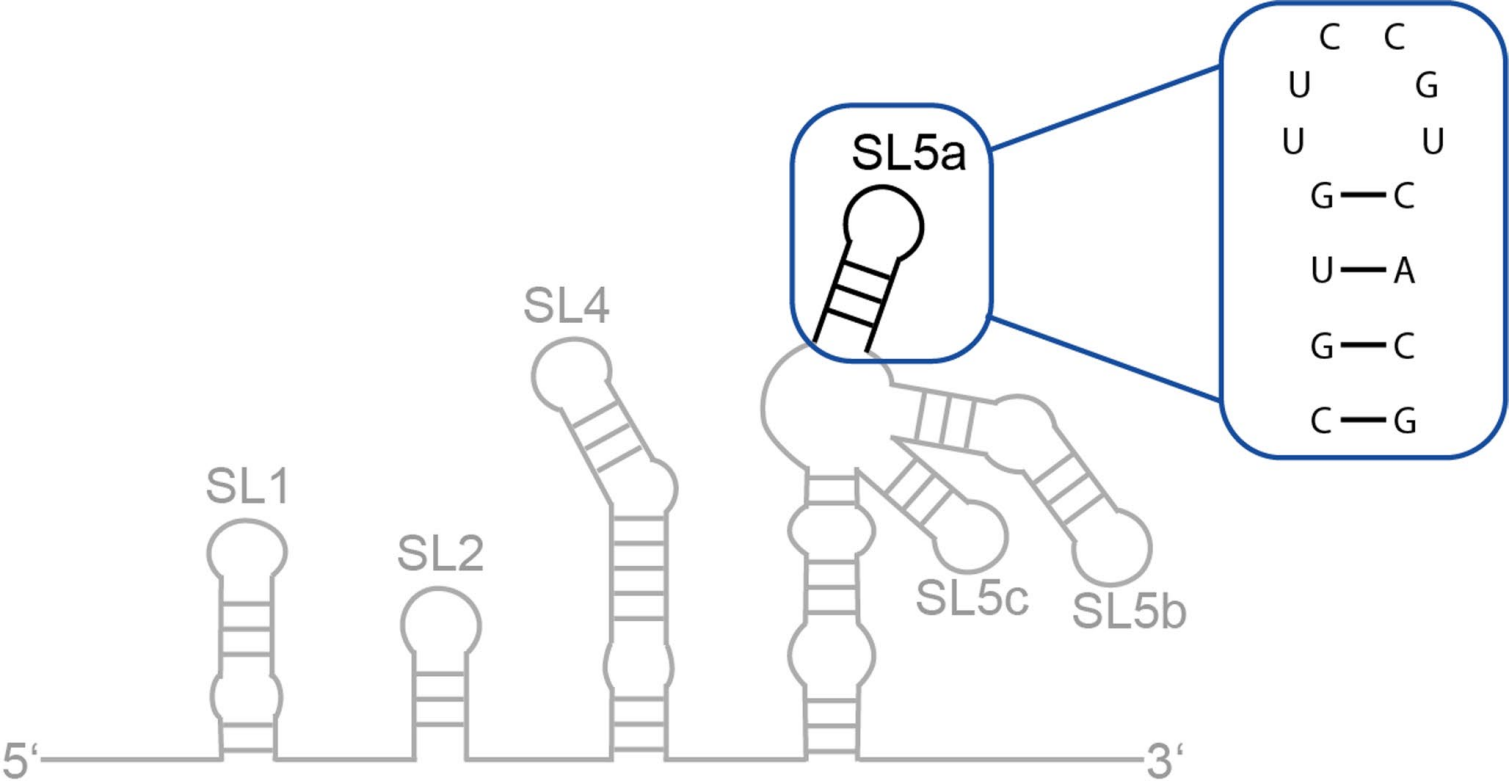
Schematic overview of the 5’-untranslated region (5’-UTR) of HCoV-229E with the different RNA stem loops (SL1, SL2, SL4, SL5a-c). 5’-SL5a is highlighted and the sequence indicated

In the context of the global pandemic caused by the Coronavirus disease 2019 (COVID-19), there has been increased interest in the structural and molecular biology of coronaviruses. To date, seven human pathogenic coronaviruses (Kesheh et al. 2022) are known. One of them is the human coronavirus 229E (HCoV-229E), an alphacoronavirus of the subgenus *Duvinacovirus* of the subfamily *Orthocoronavirinae*. Like SARS-CoV-2 (SCoV-2), it has a single-stranded (+) RNA genome of approximately 30 kb (Madhugiri et al. 2014; Chen et al. 2021). The 5’-untranslated region (5’-UTR) is crucial for viral replication and transcription and structurally conserved to a different extent across different coronavirus genera. It is proposed to fold into four to five structural stem loops (SLs) depending on the respective coronavirus. In most betacoronaviruses, five SL structures (SL1, SL2, SL3, SL4 and SL5) are found in the 5’-UTR, while alphacoronaviruses fold into four conserved SL structures (SL1, SL2, SL4 and SL5; Fig. 1) (Madhugiri et al. 2014, 2024). The conserved 5’-UTR SL5 (5SL5) structural element contains three substructured hairpins, named SL5a, SL5b, and SL5c (Chen and Olsthoorn 2010; Madhugiri et al. 2014). It has been suggested that SL5 has a role in genome packaging (Masters 2019; Chen et al. 2021; Chechetkin and Lobzin 2022).

Recent studies have shown that 5SL5 adopts a complex fold, including a T-shaped four-way junction with two large stem loops, a smaller stem loop and an additional stem (Kretsch et al. 2024; de Moura et al. 2024). High-resolution NMR structures of the two large stem-loops of SCoV-2 SL5a and SL5b confirm the presence of a conserved repetitive structural motif (RSM), UUYCGU, capping the two large loops (Chen et al. 2021; Mertinkus et al. 2024). This motif is essential for maintaining RNA stability and function. Notably, the AUG start codon of the main protein synthesis open reading frame (ORF) is embedded within the largest stem of 5SL5, highlighting its functional importance in translational regulation (Kretsch et al. 2024; de Moura et al. 2024).

Although 5SL5 is a common structural element of HCoV-229E and SCoV-2, there are significant differences. In alphacoronaviruses such as HCoV-229E, RSMs are highly conserved, with the sequence 5’-UUCCGU-3’ predominant in all of the three hairpins (SL5a, b and c), whereas in betacoronaviruses such as SCoV-2, sequence variation is more diverse, with motifs such as 5’-UUUCGU-3’ and 5’-UUUUGU-3’, or even completely different motifs in members of the subgenus *Embecovirus* (BCoV, MHV, HCoV-OC43). These differences may affect RNA folding dynamics, interactions with host proteins, and genome packaging (Madhugiri et al. 2014; Chen et al. 2021).

Evaluating how these sequence variations translate into structural features of SL5 across coronaviruses allows us to assess how these elements contribute to viral RNA synthesis, translation regulation, and host-virus interactions. NMR spectroscopy enables the investigation of conformational and dynamic features of RNA in solution, and is therefore a powerful tool to probe RNA secondary structure. To provide a more detailed insight into the structure of 5SL5a from HCoV-229E and to allow comparisons with SCoV-2, we here present a ^1^H, ^13^C and ^15^N chemical shift assignment of the wildtype (WT) SL5a from HCoV-229E and various loop mutants. Such chemical shift assignments (Wacker et al. 2020; Schnieders et al. 2021; Richter et al. 2021; Vögele et al. 2021; Mertinkus et al. 2022; Matzel et al. 2024; Maria et al. 2025) are the essential requirement for subsequent RNA structure determination (Vögele et al. 2023, 2024; Toews et al. 2024; Mertinkus et al. 2024), which can uniquely be obtained by high-resolution, heteronuclear, multidimensional NMR spectroscopy.

## Methods and experiments

### Sample preparation

Unlabelled RNAs were purchased by Horizon Discovery for NMR experiments and CD spectroscopic investigations. The purchased RNA was purified following the manufacturer’s instructions. Isotope labelled RNA for further NMR experiments was produced in house by T7-based in-vitro transcription following the description of Wacker, Weigand et al. (Wacker et al. 2020), as described in the following:

For T7-based in-vitro transcription of 5SL5a, linearised DNA template was used. An HDV ribozyme-encoding pSP64 plasmid (Promega) with a T7 promoter was used and the target sequence derived from annealed primers was inserted between the *EcoRI* and *NcoI* sites.

The recombinant vector was transformed in *E. coli* DH5α strain, and plasmid DNA (2–10 mg/L SB medium) was purified by Gigaprep (Qiagen) following the manufacturer’s protocol, linearised with *HindIII*, and transcribed using T7 RNA polymerase (P266L mutant) (Guillerez et al. 2005). Preparative-scale transcriptions (10–15 mL each) were performed. Transcriptions were terminated with EDTA and precipitated with 2-propanol. For RNA isolation, PAGE (polyacrylamide gel electrophoresis) with 15–17% denaturing PAA (polyacrylamide) were performed at 240 V for 5-6 h. Fragments were visualised by UV shadowing, excised and eluted into 0.3 M NaOAc by freezing at -80 °C for 30 min, a subsequent heat shock at 65 °C and shaking at 1300 rpm overnight. The supernatant was precipitated with 2-propanol. Residual PAA was removed by reversed-phase HPLC using a Kromasil RP 18 column with a 0–40% acetonitrile/triethylammonium acetate gradient. RNA fractions were freeze-dried, further purified via LiClO_4_ precipitation (2% in acetone), and folded by heating to 80 °C followed by rapid cooling.

Buffer exchange into NMR buffer (50 mM potassium chloride, 25 mM potassium phosphate, pH 6.2) was done using centrifugal concentrators (2 kDa cut-off). RNA purity was verified by denaturing PAA gel electrophoresis.

A *cis*-acting 3’-HDV ribozyme processed T7-transcribed RNAs, which consequently contain a 2’,3’ cyclic phosphate group at their 3’-termini (nucleotide C18). In contrast, chemically synthesised RNAs have a 3’-OH group. The differences at the 3’-terminus lead to differences in chemical shifts for the 3’-terminal nucleotide.

**Fig. 2:**
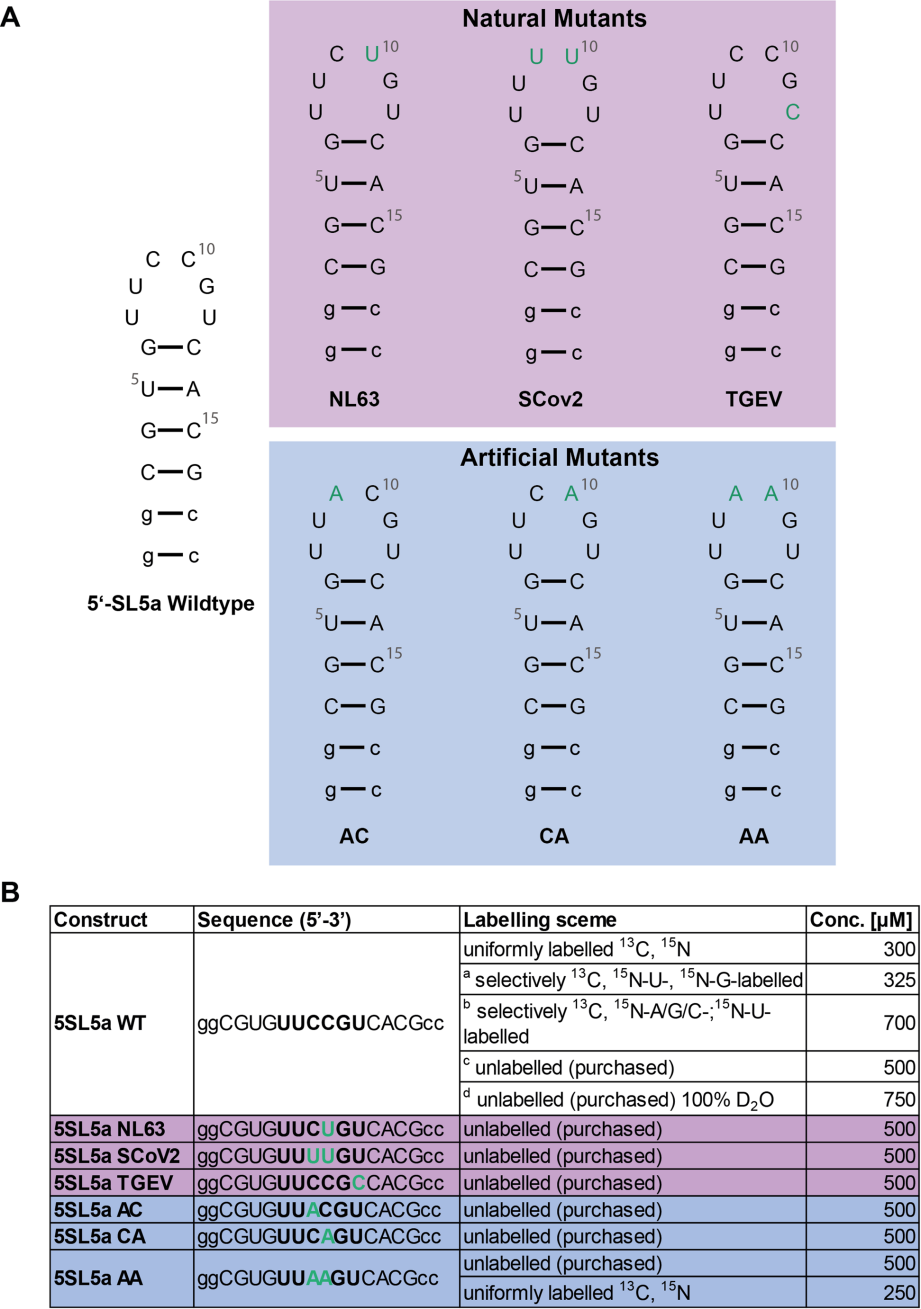
Isolated sequences of the SL5a wildtype (WT) and six loop mutations for biophysical investigations of loop dynamics. The mutated nucleotides are annotated in green. Mutations identified in sequencing of the viral genome mutants are highlighted in pink, artificial mutations introduced for the biophysical studies conducted here are highlighted in blue. Non-native bases are written in lower case letters. **A)** Secondary structures of the sequences. **B**) Table of the sequences. The apical loop is marked bold, mutated bases are written in green. Different labelling schemes that were used for the resonance assignment and sample concentrations are listed

The nucleotides 186 to 199 (14nt) from the SL5a RNA are used with the native loop sequence (Madhugiri et al. 2014) and six different loop-sequence variations (hexaloop structures) (Fig. 2). Two non-native GC basepairs were added to each construct for the homogeneous T7-based RNA production. For the purposes of simplicity, the nucleotide annotations have been adjusted to start at 1, with the numbers counting up.

### Circular dichroism spectroscopy and thermal melting curve analysis

To define the stability of the different stem loop sequences, melting points were determined by circular dichroism spectroscopy. Measurements were performed with a Jasco J-810 spectropolarimeter and recorded from 200 to 320 nm at 298 K using a 1 mm path-length quartz cuvette (Hellma 110-QS, Hellma GmbH) with 8 µM RNA for each samples in 220 µL.

Spectra were acquired with eight accumulation scans at a speed of 50 nm/min and at 25 °C. Baselines were corrected and smoothed with Savitzky-Golay filters (15 fit). Measured ellipticities were recorded in millidegrees (mdeg) and converted to molar ellipticity [θ] = deg×cm^2^×dmol^-1^. Melting curves were measured at constant wavelength (maxima of the CD spectra) with varying temperature ranging from 278 K to 368 K and a sampling rate of 1 K/min. All data were analysed using Microsoft Excel 2016 and SigmaPlot 12.5. Melting temperature data were converted to normalized ellipticity and analysed in SigmaPlot using the following equation (Leisegang et al. 2022) with *x*_*0*_ and *x*_*2*_ for the first and second melting point, *a* and *c* for the amplitude of the first and the second transition and *b* and *d* the slope 1 and 2:

1 $$\:\varvec{f}=\frac{\mathbf{a}}{(1+\mathbf{exp}(-\frac{\left(\varvec{x}-\varvec{x}0\right)}{\varvec{b}}\left)\right)}+\:\frac{\mathbf{c}}{(1+\mathbf{exp}(-\frac{\left(\varvec{x}-\varvec{x}2\right)}{\varvec{d}}\left)\right)}$$.

### NMR spectroscopy experiments

All NMR experiments were performed at the BMRZ (Center for Biomolecular Magnetic Resonance) of Goethe University Frankfurt using Bruker spectrometers equipped with following consoles/ probes: 600 MHz (AVIII/ 5 mm RT TXI ^1^H [^13^C,^15^N), 600 MHz (AVNEO/ 5 mm Cryo TCI ^1^H, [^13^C,^15^N]), 599 MHz (AVNEO/ 5 mm Cryo TCI ^1^H, [^13^C,^15^N]), 600 MHz (AV NEO/ 1.7 mm Cryo TCI ^1^H [^13^C,^15^N]), 800 MHz (AVIII/ 5 mm Cryo TXO ^13^C[^1^H,^15^N]). Experiments were conducted at a temperature of 298 K (Table SI 1; Table SI 2) except for the temperature series for the 1D ^1^H-JR, where temperature was varied between 278 K and 308 K. Experiments included ^1^H,^1^H-NOESY, ^1^H,^1^H-TOCSY, ^1^H,^15^N-BEST-TROSY,^1^H,^15^N-HSQC, ^1^H,^15^N-HNN-COSY, ^1^H,^13^C-HSQC, ^1^H,^13^C-HCCNH, 3D ^1^H,^13^C-NOESY-HSQC, 3D-CNC and 3D-H(C)N (Table SI 1; Table SI 2). The processing of the NMR spectra was conducted using Topspin (version 4.3.0 and 4.4.0), while the resonance assignment was performed using nmrfam Sparky 1.470 (Lee et al. 2015). Sodium trimethylsilylpropanesulfonate (DSS) was used for the calibration of spectra to ensure correct determination of chemical shifts of ^1^H, while ^13^C and ^15^N were indirectly referenced as described by (Wishart et al. 1995).

### {^1^H},^13^C heteronuclear NOE analysis

{^1^H},^13^C heteronuclear NOEs (hetNOEs) were determined using constant-time evolution in the ^13^C dimension, employing interleaved acquisition with and without NOE buildup, and incorporating temperature compensation to ensure temperature stability. The inter scan delay was set to 5 s and the number of scans was set to 36. The values were calculated by the equation hetNOE= (I_sat_/I_ref_). Errors were calculated based on the signal/noise ratios (Farrow et al. 1994; Mertinkus et al. 2024).

### Extent of chemical shift assignments and data deposition

**Fig. 3:**
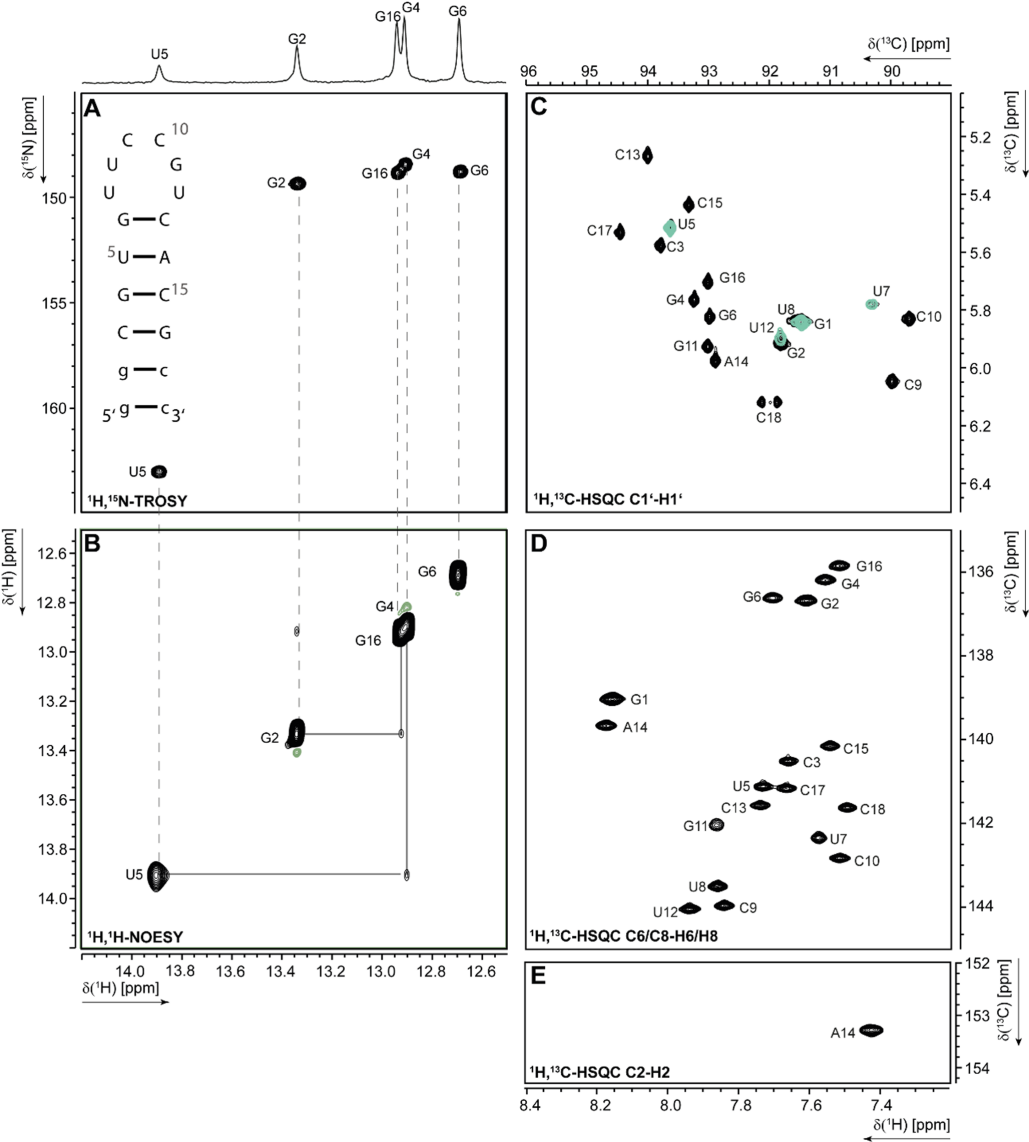
Imino and aromatic region assignment of the 5SL5a WT construct at 298 K using **A)** ^1^H,^15^N-TROSY and the secondary structure of the SL5a WT RNA, **B)** ^1^H,^1^H-NOESY, **C)** ^1^H,^13^C-HSQC for the C1’-H1’ region overlay uniformly ^13^C,^15^N-labelled RNA (black) and selectively ^13^C,^15^N-U-labelled RNA (turquoise), **D)** ^1^H,^13^C-HSQC for the C6/C8-H6/H8 region and **E)** ^1^H,^13^C-HSQC for the C2-H2 region. Imino-proton correlations in **B** are visualized by grey lines. Positive contours are given in black, negative contours in light green. Detailed experimental information can be found in Table SI 1

The imino, aromatic and ribose resonances were assigned using a uniformly ^13^C, ^15^N-labelled, a selectively ^13^C, ^15^N-U-labelled + ^15^N-G-labelled, a selectively ^13^C, ^15^N-A/G/C-labelled + ^15^N-U-labelled and an unlabelled purchased RNA sample. The experiments used for the assignment of the SL5a WT construct are listed in Table SI 1. Initially, the imino proton resonances were assigned using ^1^H,^15^N-BEST-TROSY (Fig. 3A) and ^1^H,^1^H-NOESY (Fig. 3B) spectra at 298 K. The stem could be fully assigned except for the terminal G1 due to fast exchange of its imino proton with solvent water.

NMR signals observed in the ^1^H,^13^C-HSQC and ^1^H,^1^H-TOCSY experiments were used to correlate H5-H6 resonance (Figure SI 1D) assignment of the uridines and cytidines and thus leading to the corresponding carbon resonances of C5 (Figure SI 1A) and C6 (Fig. 3D). The ^1^H,^1^H-TOCSY spectra were measured using both IVT-prepared and solid-phase synthesised RNA, which led to shifts in the resonances of the terminal 3’-nucleotide C18. This shift was caused by the different 3’-termini: a 3’-OH group in the case of the chemically synthesised RNA and a 2’,3’-cyclic phosphate group in the case of the T7-transcribed RNA (Figure SI 1D). Using a ^1^H,^13^C-HCCNH experiment (Figure SI 1 C), cross peaks were observed between guanosine H1-C8 atoms, leading to the assignment of C8-H8 resonances of the base paired nucleotides by their correlation with the guanosine H1 imino proton. The assignment of the remaining C6-H6/C8-H8 resonances was achieved using 3D-NOESY-HSQC and ^1^H,^1^H-NOESY spectra. The latter experiments were also used to determine the C1’-H1’ chemical shifts (Fig. 3C). Overlapping uridine signals were separately assigned with the selectively ^13^C, ^15^N-U-labelled sample to complete the assignment of the C1’-H1’ resonances.

The ^13^C-detected HCNC (Figure SI 1B) of the ^13^C,^15^N-A/G/C-labelled RNA was used to support the cytidine and guanosine aromatic C1’-C6/C8 signal assignment. Additionally, the C2-H2 of A14 was assigned using ^1^H,^13^C-HSQC (Fig. 3E). ^1^H,^15^N-HCN (Figure SI 2 A) and ^1^H,^15^N-HSQC (Figure SI 2B) and ^1^H,^15^N-HNN-COSY (Figure SI 2 C) were used for the assignment of H6/H8-N1/N9 and H1’-N1/N9 resonances, as well as H8-N7/N9 resonances of adenosine and guanosine. In summary, we assigned 25% of the exchangeable (^1^H-^15^ N) and 100% of the non-exchangeable base resonances (^1^H-^13^ C) as well as 60% of the ribose resonances. Additionally, we assigned 100% of the N7/N9/N1 resonances.

### The 5’-UUCCGU-3’ hexaloop

The SL5a WT features the highly conserved RSM motif 5’-UUCCGU-3’ hexaloop. The assignments of the aromatic loop resonances were derived from sequential contacts from H6/H8 to H5 and H1’ to H6/H8 using 3D-NOESY-HSQC, ^1^H,^1^H-NOESY (aqueous sample and 100% D_2_O) and ^1^H,^1^H-TOCSY spectra and correlating ^1^H,^13^C-HSQC (Fig. 3C, D,E; Figure SI 1 A, D). The overall resonance assignment of the hexaloop 5’-UUCCGU-3’ of HCoV229E showed characteristic resonances similar to the 5’-UUUCGU-3’ hexaloop SL5a from SCoV2 (Wacker et al. 2020; Schnieders et al. 2021), confirming the structural similarity of the two different coronavirus RSMs.

**Fig. 4:**
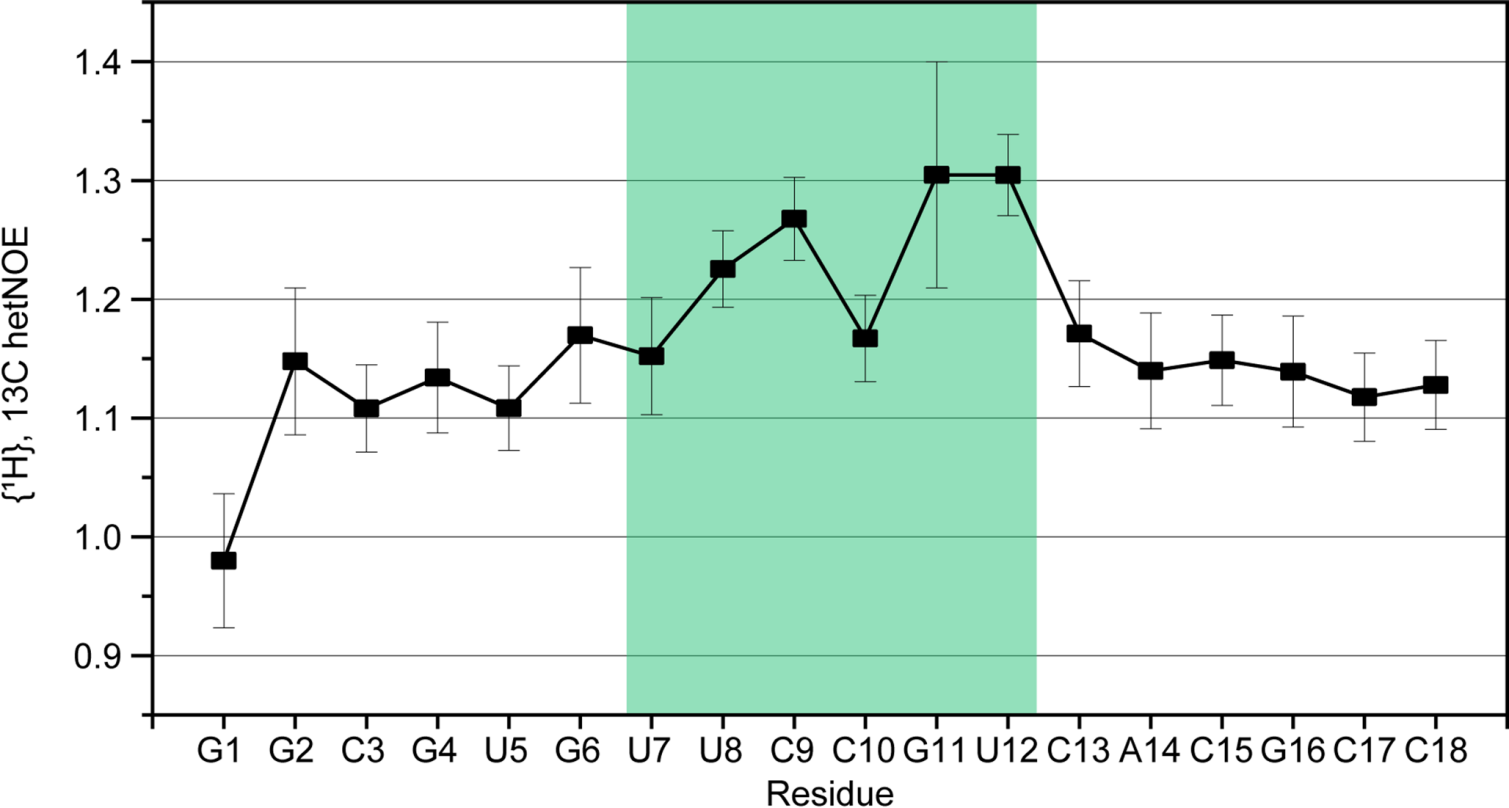
hetNOE values for aromatic CH bonds (C6-H6/C8-H8) of the SL5a WT. Measurements performed at 298 K. Loop region is highlighted in green. For {^1^H},^13^C hetNOEs, increased dynamics are indicated by higher hetNOE ratios

Analysis of heteronuclear {^1^H},^13^C-NOEs (hetNOE) of the aromatic resonances revealed an overall rigid stem with hetNOE values around 1.1, while the loop residues mainly showed increased dynamics for the C6H6/C8/H8 resonances, which is demonstrated by increased hetNOE ratios for {^1^H},^13^C hetNOEs. The largest hetNOE values of 1.3 can be detected for G11 and U12, closely followed by C9 with a hetNOE value of 1.27 (Fig. 4).

### Comparison of the wildtype to the natural loop variants

**Fig. 5:**
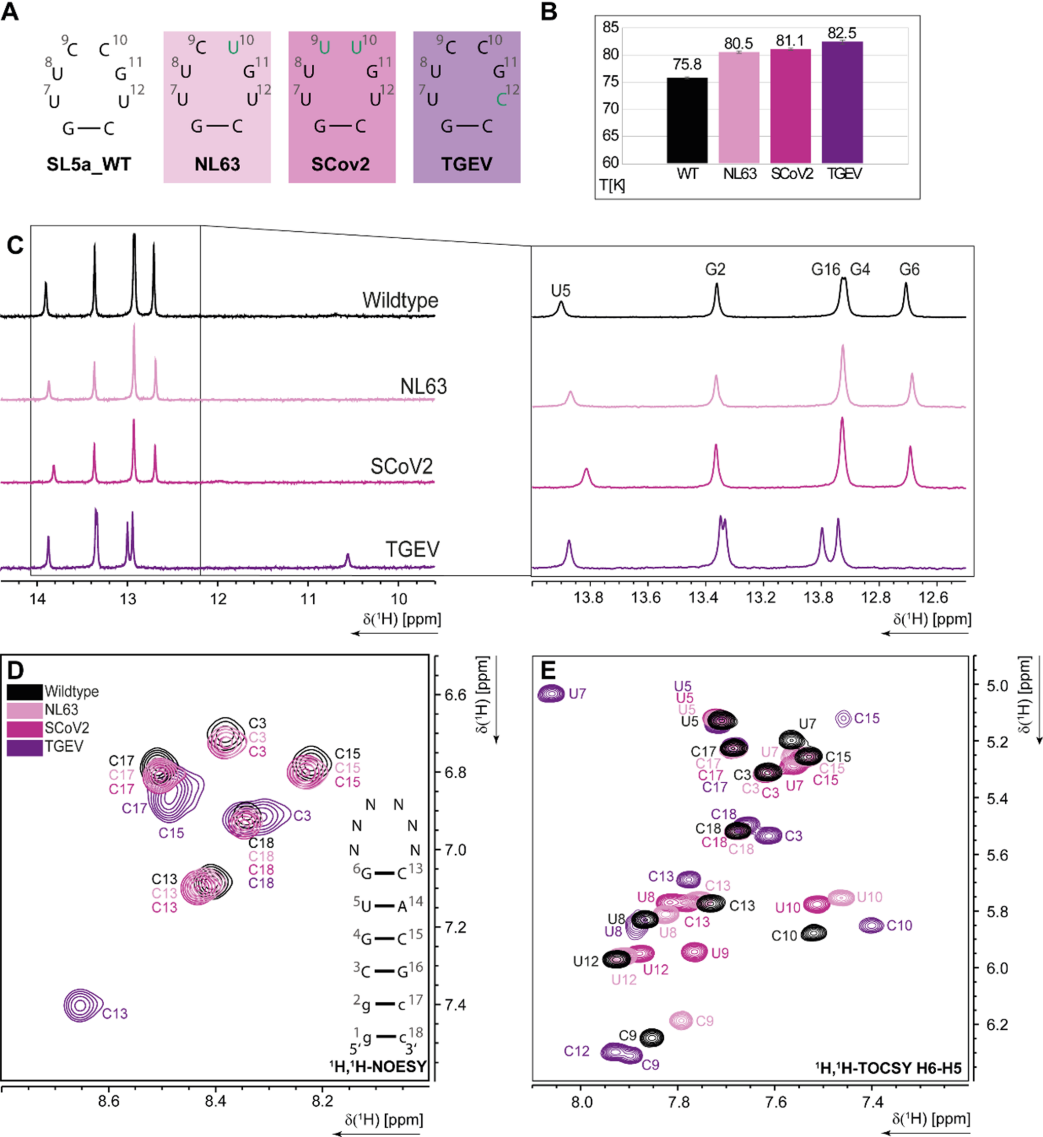
**(A)** Hexaloop sequences of the different constructs, mutated bases are indicated in blue. **(B)** Melting points of the different constructs determined by CD spectroscopic melting analysis. **(C)** ^1^H-1D NMR spectra of the imino region, **(D)** ^1^H,^1^H-NOESY amino proton region overlay of the 5SL5a WT and natural occurring loop variations NL63 (light pink), SCoV2 (magenta) and TGEV (violet) at 298 K. Secondary structure of the stem loop of the constructs is shown in the right corner. Imino proton assignment from the SL5a WT is indicated in **C)** and **D)**. **(E)** ^1^H,^1^H-TOCSY H5-H6 region overlay of the 5SL5a WT and natural occurring loop variations NL63 (light pink), SCoV2 (pink) and TGEV (purple) at 298 K. WT assignment is indicated

In the next step, we compared the NMR data of SL5a WT to other naturally occurring loop RSMs. Loop variations belonging to the HCoV-NL63 (C10U), TGEV (U12C) and a prevalent SCoV-2 (C9U, C10U) sequence (Fig. 5A) were investigated and revealed different structural features and altered dynamics depending on the RNA sequence.

While the imino proton patterns of the mutants NL63 and SCoV2 were very similar to the WT spectra, TGEV showed larger changes in the imino area, where the G6 signal shifts prominently. Besides that a new imino signal in the non-canonical region appeared around 10.6 ppm (Fig. 5C). In line with that, an increase in melting point (by almost 7 °C) from 75.8 °C to 82.5 °C was observed, suggesting that the U12C transition in the loop stabilized the TGEV SL5 compared to the HCoV-229E RNA. The melting points of the other constructs were also higher than for the WT, but less pronounced than TGEV (Fig. 5B, Figure SI 3 A, B). Chemical shift changes were detected in the amino area of ^1^H,^1^H-NOESY and H5-H6 area of ^1^H,^1^H-TOCSY spectra (Fig. 5D, E). While the H42-H41 signals of the stem cytosines of the NL63 and SCoV2 mutants were largely consistent with the WT, more pronounced changes are evident in TGEV, where C3, C13 and C15 reveal significant shifts (Fig. 5D). For the NL63 and SCoV2 loop variants, the H5-H6 signals of the stem were similar to the WT. For the loop signals, small chemical shift differences were detectable, most prominent for C10 which is changed to a U in both sequences. These small changes show that the overall structure remains largely the same, only the nucleotide identity leads to slightly altered chemical environment. For these two constructs, the assignment could be easily transferred from the WT. In contrast, the TGEV construct showed significantly larger chemical shift changes (Fig. 5E). Here, also the stem signals were shifted compared to the WT, indicating more pronounced changes in structure and stability, as evidenced by the altered melting temperature of the TGEV RNA. To distinguish between U and C signals, natural abundance HSQCs of the C5-H5 area were recorded to assign the signals in the ^1^H,^1^H-TOCSY spectra. U7 was shifted prominently in the TGEV construct, which indicates the transient base-pairing character of U7-U12. The strong shift there was hence attributed to the change of the “binding partner”, U12 to C12 (Fig. 5E (purple)).

These loop sequence comparisons show how different positions in the respective hexaloops can cause varying degrees of impact on the structural and dynamic behaviour of the whole SL5a construct. While the change of a pyrimidine base to another pyrimidine base (transition) at position 9 and 10 did not show severe impacts on the RSM, the exchange of U12 to C had strong impact on the SL5a RSM.

### Comparison of the wildtype to the artificial loop variants

**Fig. 6:**
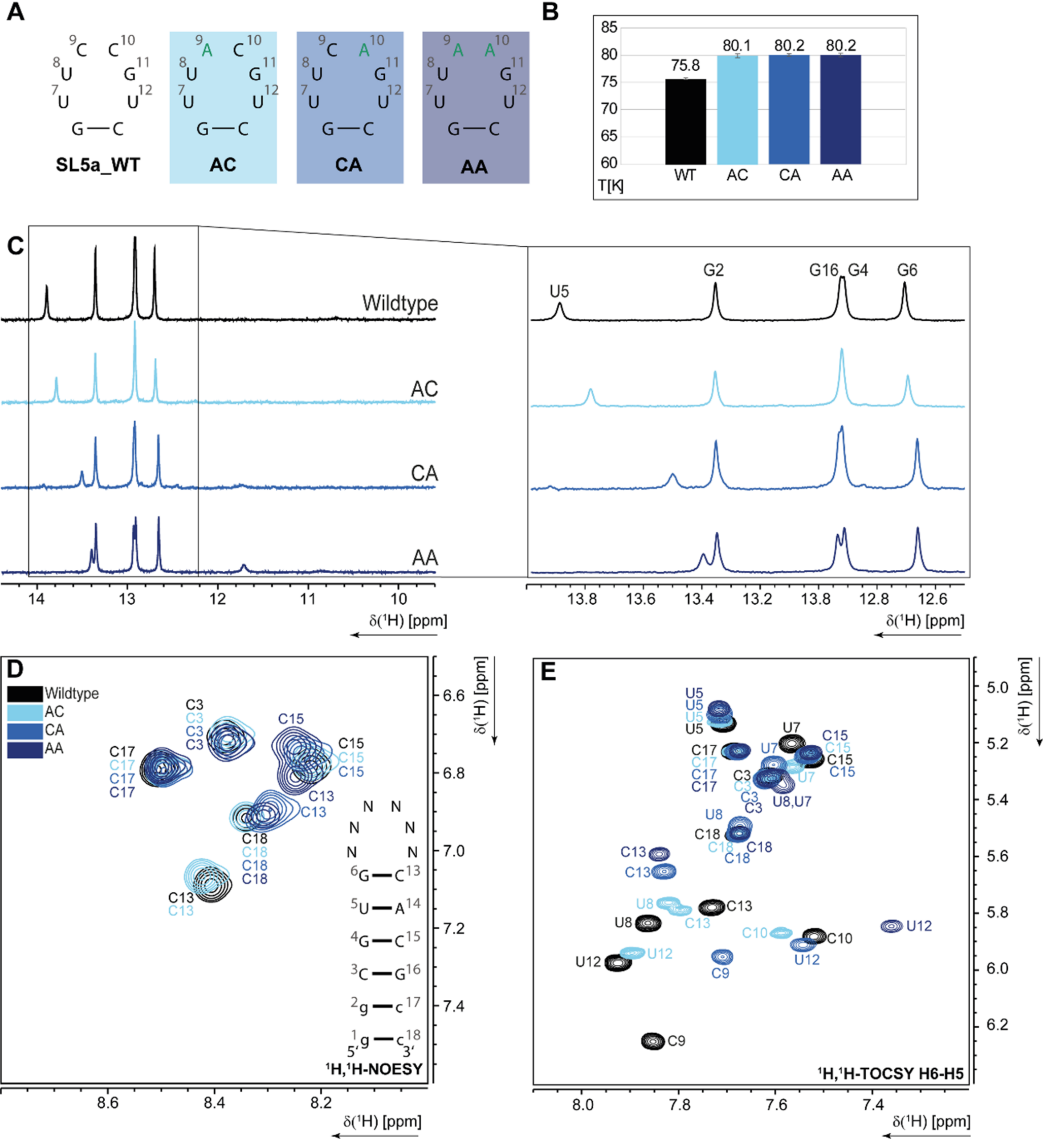
**(A)** Hexaloop sequences of the different constructs, mutated bases are indicated in blue. **(B)** Melting points of the different constructs determined by CD spectroscopic melting analysis. **(C)** ^1^H-1D NMR spectra of the imino region, **(D)** ^1^H,^1^H-NOESY amino proton region overlay of the 5SL5a WT and artificial loop variations AC (light blue), CA (blue) and AA (dark blue) at 298 K. Secondary structure of the stem loop of the constructs is shown. Imino proton assignment from the SL5a WT is indicated in **C)** and **D)**. **(E)** ^1^H,^1^H-TOCSY H5-H6 region overlay of the 5SL5a WT and natural occurring loop variations NL63 (light blue), SCoV2 (blue) and TGEV (dark blue) at 298 K. WT assignment is indicated in black, suggested assignment of the mutants are indicated in light blue (AC), blue (CA) and dark blue (AA)

Additionally, SL5a WT was compared to artificial loop mutations, where C9 and/or C10 were changed to adenosine, leading to differential effects on the overall structure (Fig. 6A). All three mutants (C9A, C10A, and C9A/C10A) showed a slightly increased melting temperature from ~ 80 °C compared to the WT with a melting point around 76 °C (Fig. 6B, Figure SI 3 C, D ). The mutated RNA constructs only showed small changes in the imino proton patterns, where U5 shifted up field, especially in the CA and AA mutants (Fig. 6C). For the AC mutant, consistently, also the amino area of ^1^H,^1^H-NOESY spectra remained nearly the same compared to the WT (Fig. 6D, black and light blue). In contrast, the CA and AA mutants exhibited stronger chemical shift variations for C13 and C15 (Fig. 6D, black, blue and dark blue). The AC loop also showed only slight shifts of the loop signals in the ^1^H,^1^H-TOCSY, most prominent for U8 and C10, the neighbouring signals of mutated A9, but the change from a pyrimidine base to a purine base at this position did not show any effect on the stem (Fig. 6E, black and light blue). However, changing C10 to A led to significant shifts of the H5-H6 signals in the ^1^H-^1^H TOCSY (Fig. 6E). While most of the stem signals remained similar to the WT, C13 of the closing base pair showed larger signal shifts. The loop signals of the CA mutants also showed significant shifts, most prominent for C9 and U12. These shifts indicate structural and/or dynamic changes of the SL5a RSM due to the mutation at position 10. These observations were consistently made also for the AA mutant where C9 and C10 are mutated to A9 and A10 (Fig. 6E black and dark blue). Here, the aforementioned effects for the CA mutant were even larger. For the AA construct, also the C13 shifts were considerable, but most prominent were the changes of the loop signals. All three loop Us showed tremendous changes, leading to the conclusion that the mutations affected structure, stability and dynamics of the SL5a RSM. While the C10U mutation only led to minor shifts as described in the previous section, the change of a pyrimidine base to a purine base had strong impact.

## Conclusion & outlook

We here present the chemical shift assignment of alpha-SL5a containing the highly conserved RSM 5’-UUCCGU-3’. We compared the naturally occurring sequence variations within these RSMs that are strictly maintained in apical hexaloops among different alpha- and betacoronaviruses. We show that although certain substitutions at certain positions are well-tolerated, most structural and, importantly dynamical features of the RSM remain conserved. Allowed substitutions exclusively comprise pyrimidine-pyrimidine transitions at positions 3, 4, and 6 of the RSM-containing hexaloops. Our designed RSM-variants exhibit the following properties: while changing position 3 and 4 of the hexaloop from one pyrimidine to another pyrimidine (transition), only small changes are detectable for the loop signals. However, a U-to-C transition at position 6 leads to a stronger impact on the overall structure and dynamics. Interestingly, changing the pyrimidine on position 3 to a purine base (transversion) does not show significant changes, but for C-to-A transversions either at position 4 or at both positions, 3 and 4 combined, severe changes in structure and dynamics are visible, demonstrating that the pyrimidines at 4 and 6 are key determinants of the SL5a hexaloop structural dynamics. Our work dissects the evolutionary constraints on viral RNA sequence, structure, and dynamics and should be regarded as a contribution towards a better understanding of the principles that govern viral evolution.

## Electronic supplementary material

Below is the link to the electronic supplementary material.

Supplementary Material 1

Supplementary Material 2

## Data Availability

The data deposition for 5SL5a WT was uploaded to BMRB. Data can be found with the code 53233. Experimental raw data have been deposited under https://doi.org/10.25716/gude.176r-eady.
