# Supplementary material for: 1H, 13C and 15N chemical shift assignment for stem-loop 5a from the 5‘UTR of HCoV-229E
Source: Biomol NMR Assign. 2025 Jul 31;19(2):255–65. doi: 10.1007/s12104-025-10243-4 (PMC12513883; doi:10.1007/s12104-025-10243-4)
Supplement: Supplementary file 1 — Supplementary Material 1 [file 12104_2025_10243_MOESM1_ESM.docx]

**Supplementary information**

for

**^1^H, ^13^C and ^15^N chemical shift assignment for stem-loop 5a from the 5‘UTR of HCoV-229E**

Nina M. Krause^1, 2^ Anna Wacker^1, 2^, Christian Richter^1, 2^, Boris Fürtig^1,2,^, Ramakanth Madhugiri^3^, John Ziebuhr^3^, and Harald Schwalbe^1, 2^

^1^ Institute for Organic Chemistry and Chemical Biology
 Johann Wolfgang Goethe University,

Max-von-Laue-Straße7, 60438Frankfurt/M., Germany

^2^ Center for Biomolecular Magnetic Resonance (BMRZ),

Johann Wolfgang Goethe University,

Max-von-Laue‑Str. 9, 60438 Frankfurt/M., Germany

^3^ Institute of Medical Virology, Justus Liebig University,
 Giessen, Germany

**Keywords**: Coronaviruses • HCoV 229E • 5‘-UTR • SL5a • Solution NMR spectroscopy


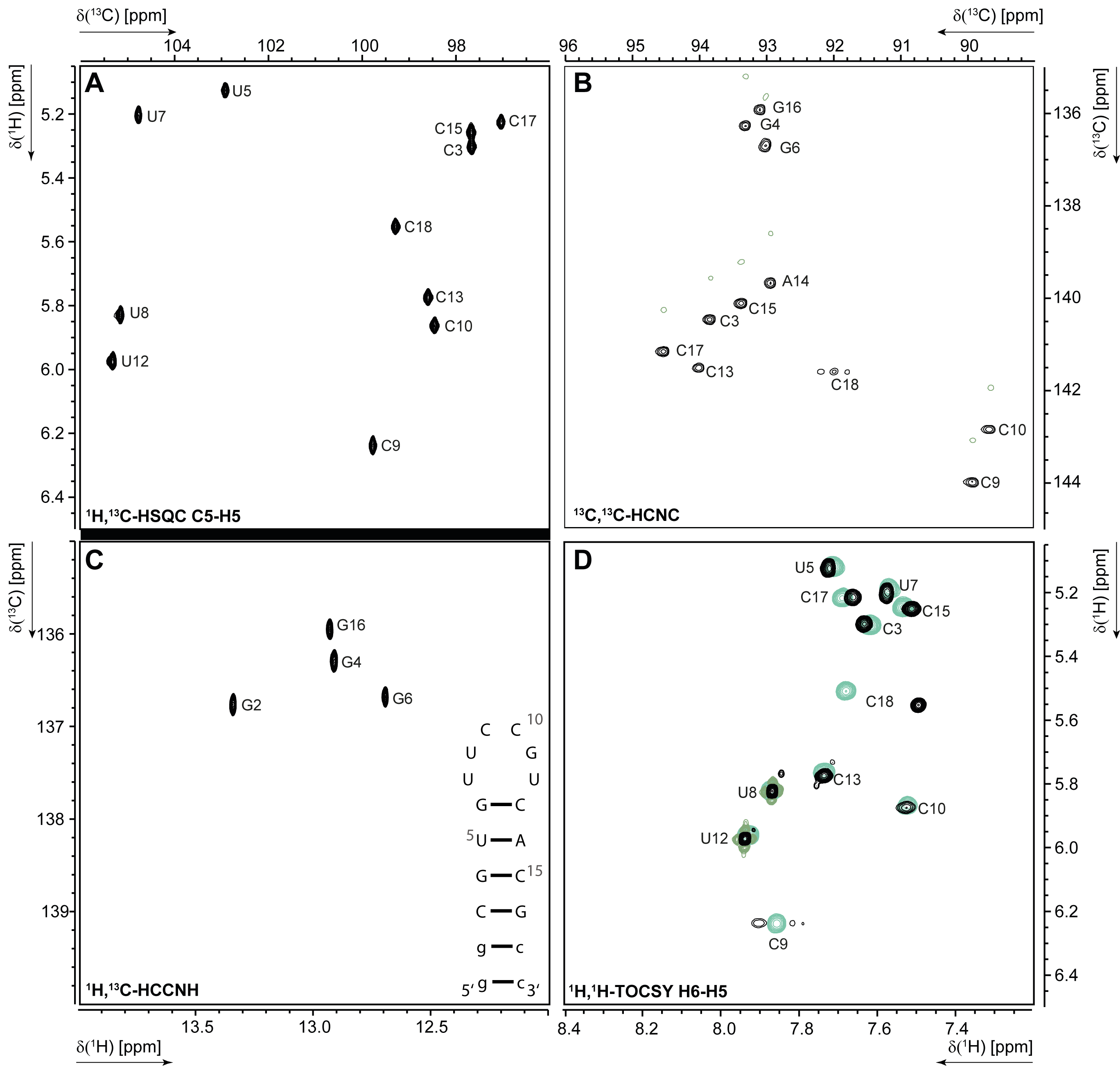


*Figure SI 1: Spectra of the 5SL5a_WT construct at 298 K.* ***A)*** *^1^H,^13^C-HSQC (C5-H5 region).* ***B)*** *^13^C,^13^C-HCNC (C6/C8-C1’ region).* ***C)*** *^1^H,^13^C-HCCNH (C6/C8-H1/H3 region).* ***D)*** *^1^H,^1^H-TOCSY (H5-H6 region), overlay IVT prepared RNA (black) and purchased RNA (turquoise).*


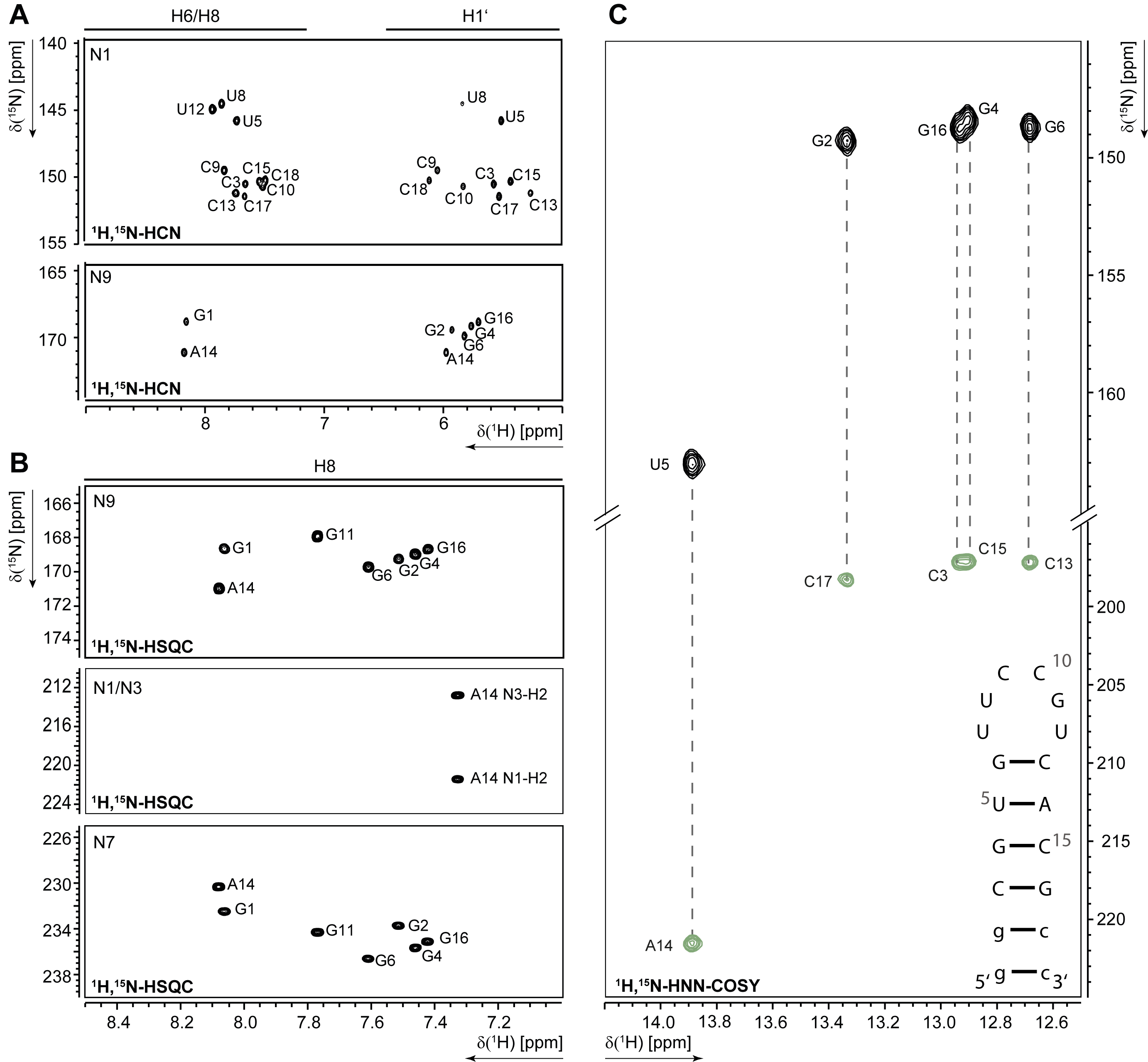


Figure SI 2: **A)** 2D plane of the ^1^H,^15^N-HCN experiment correlation the H6/H8 and H1’ resonances with the N1 and N9 resonances. **B)** ^1^H,^15^N-HSQC experiment showing the H8-N9/N7 correlations. **C)** ^1^H,^15^N-HNN-COSYand the secondary structure of the SL5a_WT RNA**.** All spectra were measured at 298 K with the uniformly ^13^C, ^15^N labelled 5SL5a_WT sample.


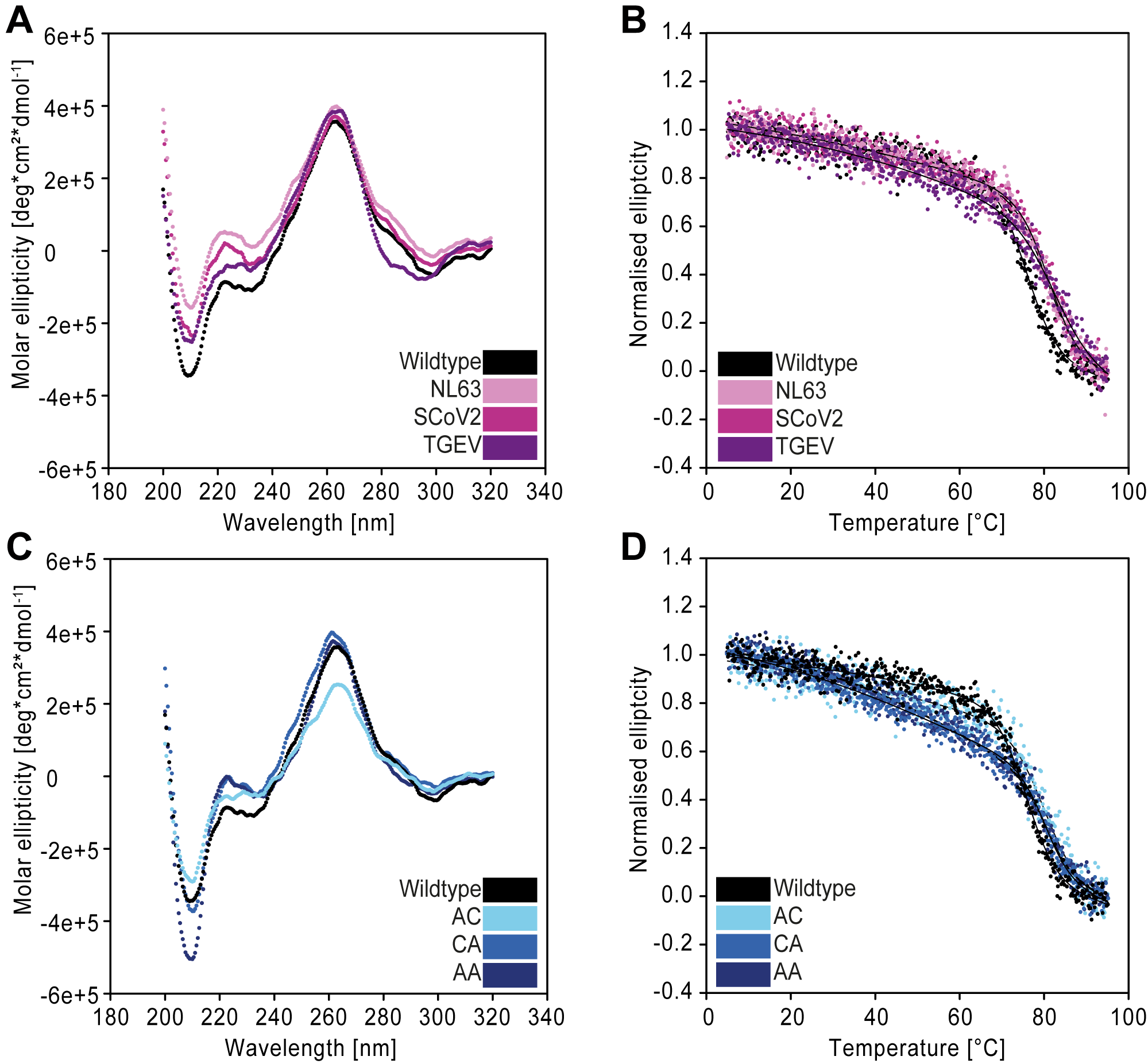


Figure SI 3: Circular dichroism-spectroscopic analysis of the different constructs, showing the variation in the stability due to different melting points. **A)** Circular dichroism spectra of the wildtype compared to the natural mutants. **B)** Thermal melting analysis of the wildtype compared to the natural mutants. **C)** Circular dichroism spectra of the wildtype compared to the artificial mutants. **D**) Thermal melting analysis of the wildtype compared to the artificial mutants. All samples had a concentration of 8 µM.
