# Supplementary material for: 1H, 13C and 15N chemical shift assignment for stem-loop 5a from the 5‘UTR of HCoV-229E
Source: Biomol NMR Assign. 2025 Jul 31;19(2):255–65. doi: 10.1007/s12104-025-10243-4 (PMC12513883; doi:10.1007/s12104-025-10243-4)
Supplement: Supplementary file 2 — Supplementary Material 2 [file 12104_2025_10243_MOESM2_ESM.docx]

**Supplementary information**

for

**^1^H, ^13^C and ^15^N chemical shift assignment for stem-loop 5a from the 5‘UTR of HCoV-229E**

Nina M. Krause^1, 2^ Anna Wacker^1, 2^, Christian Richter^1, 2^, Boris Fürtig^1,2,^, Ramakanth Madhugiri^3^, John Ziebuhr^3^, and Harald Schwalbe^1, 2^

^1^ Institute for Organic Chemistry and Chemical Biology
 Johann Wolfgang Goethe University,

Max-von-Laue-Straße7, 60438Frankfurt/M., Germany

^2^ Center for Biomolecular Magnetic Resonance (BMRZ),

Johann Wolfgang Goethe University,

Max-von-Laue‑Str. 9, 60438 Frankfurt/M., Germany

^3^ Institute of Medical Virology, Justus Liebig University,
 Giessen, Germany

**Keywords**: Coronaviruses • HCoV 229E • 5‘-UTR • SL5a • Solution NMR spectroscopy

Table SI 1: List of NMR experiments and experimental parameters used for chemical shift assignment of the 5SL5a WT. Measurements were conducted at 298 K in 90% aqueous NMR-buffer (50 mM KCl, 25 mM K_2_HPO_4_/KH_2_PO_4_, pH 6.2) + 10% D_2_O. Measurements that were not conducted with the uniformly ^13^C, ^15^N-labelled are labelled (^a^ selectively ^13^C, ^15^N-U-labelled+^15^N G; ^b^ selectively ^13^C, ^15^N-A/G/C-labelled+^15^N-labelled U; ^c^ unlabelled purchased RNA, ^d^ unlabelled purchased RNA (100% D_2_O)).

| **NMR experiments** | **Experimental parameters** | **Characteristic parameters** |
| --- | --- | --- |
| **^1^H,^1^H-NOESY**  Jump return Water suppression  (Hwang and Shaka 1995; Sklenar 1995) | 800 MHz, ns: 136, sw(f2): 21.12 ppm, sw(f1): 11.90 ppm, aq(f2): 60.6 ms, aq(f1): 20.1 ms, o1(^1^H): 4.7 ppm, o3(^15^N): 153 ppm, relax. delay: 1.0 s, time: 19 h 15 min | NOE mixing time: 250 ms, |
| **^1^H,^1^H-NOESY**  Excitation sculpting Water suppression  (Hwang and Shaka 1995; Sklenar 1995) | ^d^ 600 MHz, ns: 64, sw(f2): 20.83 ppm, sw(f1): 11.90 ppm, aq(f2): 120 ms, aq(f1): 128.6 ms, o1(^1^H): 4.69 ppm, o3(^15^N): 120 ppm, relax. delay: 1.0 s, time: 19 h 15 min | NOE mixing time: 150 ms, |
| **^1^H,^15^N-BEST-TROSY**  (Solyom et al. 2013) | 800 MHz, ns: 16, sw(f2): 21.12 ppm, sw(f1): 24.66 ppm, aq(f2): 59 ms, aq(f1): 64 ms, o1(^1^H): 4.7 ppm, o2(^13^C): 101 ppm, o3(^15^N): 153 ppm, relax. delay: 0.3 s, time: 28 min | HN transfer time: 2.7 ms ^1^J_NH_: 12.35 Hz |
| **^1^H,^13^C-HSQC**  C2-C6-C8  (Bodenhausen and Ruben 1980) | 800 MHz, ns: 4, sw(f2): 8.35 ppm, sw(f1): 23.99 ppm, aq(f2): 76.7 ms, aq(f1): 27 ms, o1(^1^H): 4.7 ppm, o2(^13^C): 143.0 ppm, relax. delay: 1 s, time: 19 min | INEPT transfer time: 1.25 ms (^1^J_CH_ 200 Hz) |
| **^1^H,^13^C-HSQC**  C5  (Bodenhausen and Ruben 1980) | 800 MHz, ns: 4, sw(f2): 8.35 ppm, sw(f1): 15.99 ppm, aq(f2): 76.7 ms, aq(f1): 39.8 ms, o1(^1^H): 4.7 ppm, o2(^13^C): 100.0 ppm, relax. delay: 1 s, time: 19 min | INEPT transfer time: 1.4 ms (^1^J_CH_ 180 Hz) |
| **^1^H,^13^C-HSQC**  C1´-H1´  (Bodenhausen and Ruben 1980) | 800 MHz, ns: 4, sw(f2): 8.35 ppm, sw(f1): 12.00 ppm, aq(f2): 76.7 ms, aq(f1): 53 ms, o1(^1^H): 4.7 ppm, o2(^13^C): 90 ppm, relax. delay: 1 s, time: 29 min  ^a^600 MHz, ns: 16, sw(f2): 8.34 ppm, sw(f1): sw(f1): 12.00 ppm, aq(f2): 102.4 ms, aq(f1): 70.7 ms, o1(^1^H): 4.7 ppm, o2(^13^C): 90 ppm, relax. delay: 1 s, time: 1 h 19 min | INEPT transfer time: 1.5 ms (^1^J_CH_ 170 Hz) |
| **^1^H,^1^H-TOCSY** | ^b^ 800 MHz ns: 16, sw(f2): 8.75 ppm, sw(f1): 6.25 ppm, aq(f2): 100 ms, aq(f1): 51 ms, o1(^1^H): 4.7 ppm, relax. delay: 1 s, time: 2 h 46 min  ^c^ 600 MHz, ns: 8, sw(f2): 8.8 ppm, sw(f1): 5.6 ppm, aq(f2): 97.2 ms, aq(f1): 57.6 ms, o1(^1^H): 4.69 ppm, relax. delay: 1 s, time: 1 h 3 min | TOCSY mixing time: 30 ms |
| **^1^H,^15^N-HSQC**  ^2^J-coupling | 600 MHz, ns: 64, sw(f2): 10 ppm, sw(f1): 78.3 ppm, aq(f2): 85.2 ms, aq(f1): 26.9 ms, o1(^1^H): 4.7 ppm, o2(^15^N): 201.5 ppm, relax. delay: 1 s, time: 2 h 36 min | HN transfer time 10 ms  ^2^J_HN_ 25 Hz |
| **^1^H,^13^C-HCCNH**  Iminos to aromatics | ^b^800 MHz, ns: 128, sw(f3): 20.9 ppm, sw(f2): 9.9 ppm, sw(f1): 30.0 ppm, aq(f3): 67.3 ms, aq(f2): 32.0 ms, aq(f1): 0.2 ms, o1(^1^H): 4.7 ppm, o2(^13^C): 137 ppm, o3(^15^N): 154 ppm, relax. delay: 1 s, time: 5 h 35 min | CC TOCSY mixing time 28 ms |
| **3D ^1^H,^13^C-NOESY-HSQC** | 800 MHz, ns: 16, sw(f3): 8.75 ppm, sw(f2): 21.00 ppm, sw(f1): 6.25 ppm, aq(f3): 73.1 ms, aq(f2): 7.5 ms, aq(f1): 16.0 ms, o1(^1^H): 4.7 ppm, o2(^13^C): 137 ppm, relax. delay: 0.9 s, time: 13 h 58 min; non uniform sampling | NOE mixing time 200 ms, |
| **3D-CNC** | ^b^ 800 MHz, ns: 16, sw(f3): 24,5 ppm, sw(f2): 34.7 ppm, sw(f1): 12.0 ppm, aq(f3): 67.5 ms, aq(f2): 5.7 ms, aq(f1): 19.9 ms, o1(^13^C): 90 ppm, o3(^15^N): 157 ppm, relax. delay: 0.5 s, time: 1 d 12 h 30 min | CN transfer time ~ 30 ms |
| **3D-H(C)N**  **2D plane** | 800 MHz ns: 64, sw(f3): 9.0 ppm, sw(f2): 16 ppm, sw(f1): 35.2 ppm, aq(f3): 70.9 ms, aq(f2): 0.15 ms, aq(f1): 33.6 ms, o1(^1^H): 4.7 ppm, o2(^13^C): 115 ppm, o3(^15^N): 157 ppm, relax. delay: 1 s, time: 4 h 8 min | HC-transfer time: 1.4 ms  CN-transfer time: 17.5 ms |
| **^1^H,^15^N-HNN-COSY** | 800 MHz ns: 64, sw(f2): 24 ppm, sw(f1): 120 ppm, aq(f2): 70.9 ms, aq(f1): 17.5 ms, o1(^1^H): 4.7 ppm, o2(^13^C): 101 ppm, o3(^15^N): 185 ppm relax. delay: 0.3 s, time: 2 h 15 min | NN-transfer time: 30 ms |

Table SI 2: List of NMR experiments and experimental parameters used for unlabelled purchased RNA samples to compare the SL5a WT and the different loop mutants. All measurements were conducted at 298 K in 90% aqueous NMR-buffer (50 mM KCl, 25 mM K_2_HPO_4_/KH_2_PO_4_, pH 6.2) + 10% D_2_O with an RNA concentration of 500 µM.

| **NMR experiments** | **Experimental parameters** | **Characteristic parameters** |
| --- | --- | --- |
| **^1^H,^1^H-NOESY**  Jump return Water suppression  (Hwang and Shaka 1995; Sklenar 1995) | 600 MHz, ns: 16, sw(f2): 20.84 ppm, sw(f1): 11.90 ppm, aq(f2): 63.3 ms, aq(f1): 35.9 ms, o1(^1^H): 4.7 ppm, relax. delay: 1.0 s, time: 29 h 49 min | NOE mixing time 150 ms, |
| **^1^H,^1^H-TOCSY** | 600 MHz, ns: 8, sw(f2): 8.8 ppm, sw(f1): 5.6 ppm, aq(f2): 97.2 ms, aq(f1): 57.6 ms, o1(^1^H): 4.7 ppm, relax. delay: 1 s, time: 1 h 3 min | TOCSY mixing time: 30 ms |


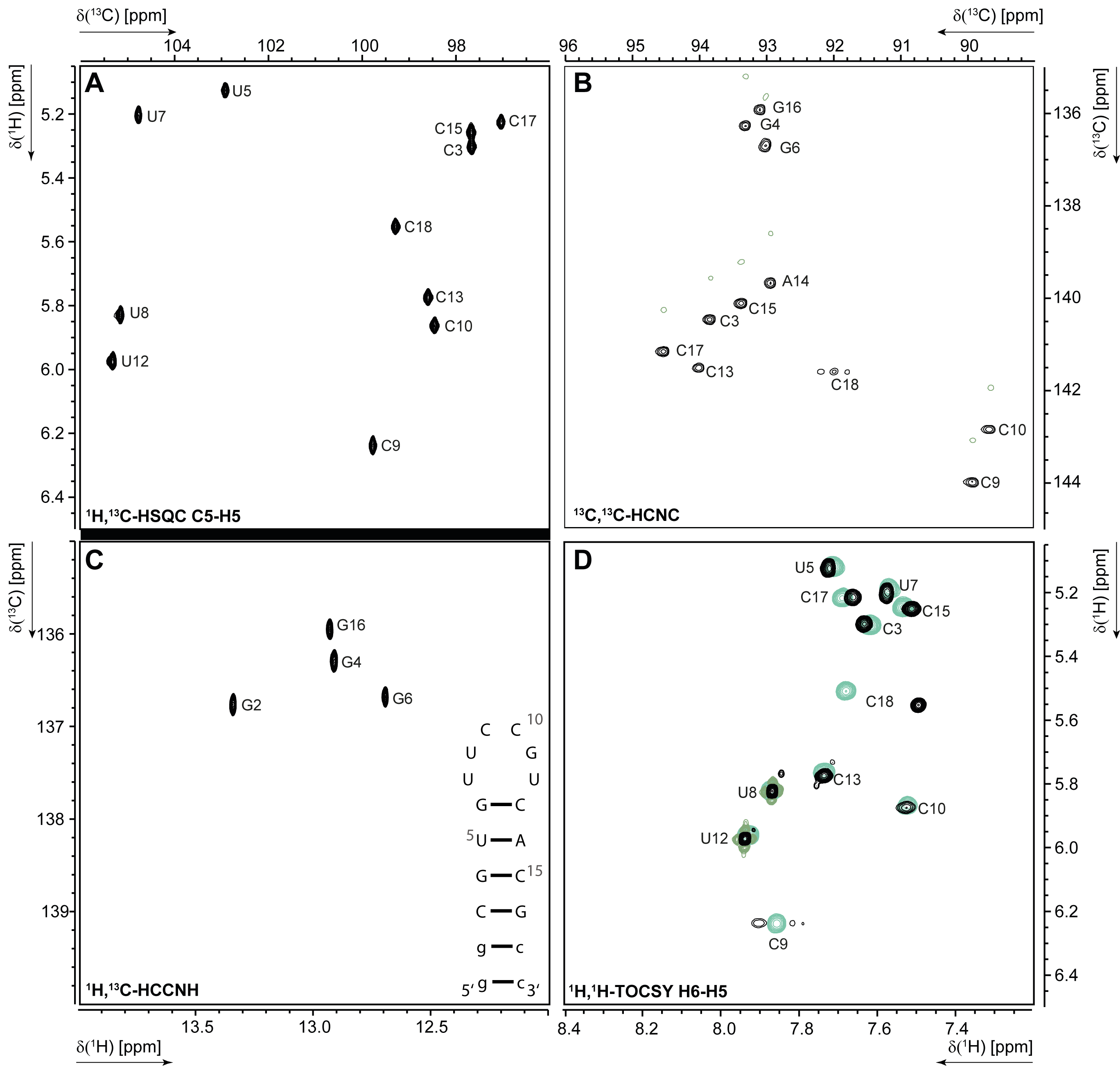


*Figure SI 1: Spectra of the 5SL5a_WT construct at 298 K.* ***A)*** *^1^H,^13^C-HSQC (C5-H5 region).* ***B)*** *^13^C,^13^C-HCNC (C6/C8-C1’ region).* ***C)*** *^1^H,^13^C-HCCNH (C6/C8-H1/H3 region).* ***D)*** *^1^H,^1^H-TOCSY (H5-H6 region), overlay IVT prepared RNA (black) and purchased RNA (turquoise).*


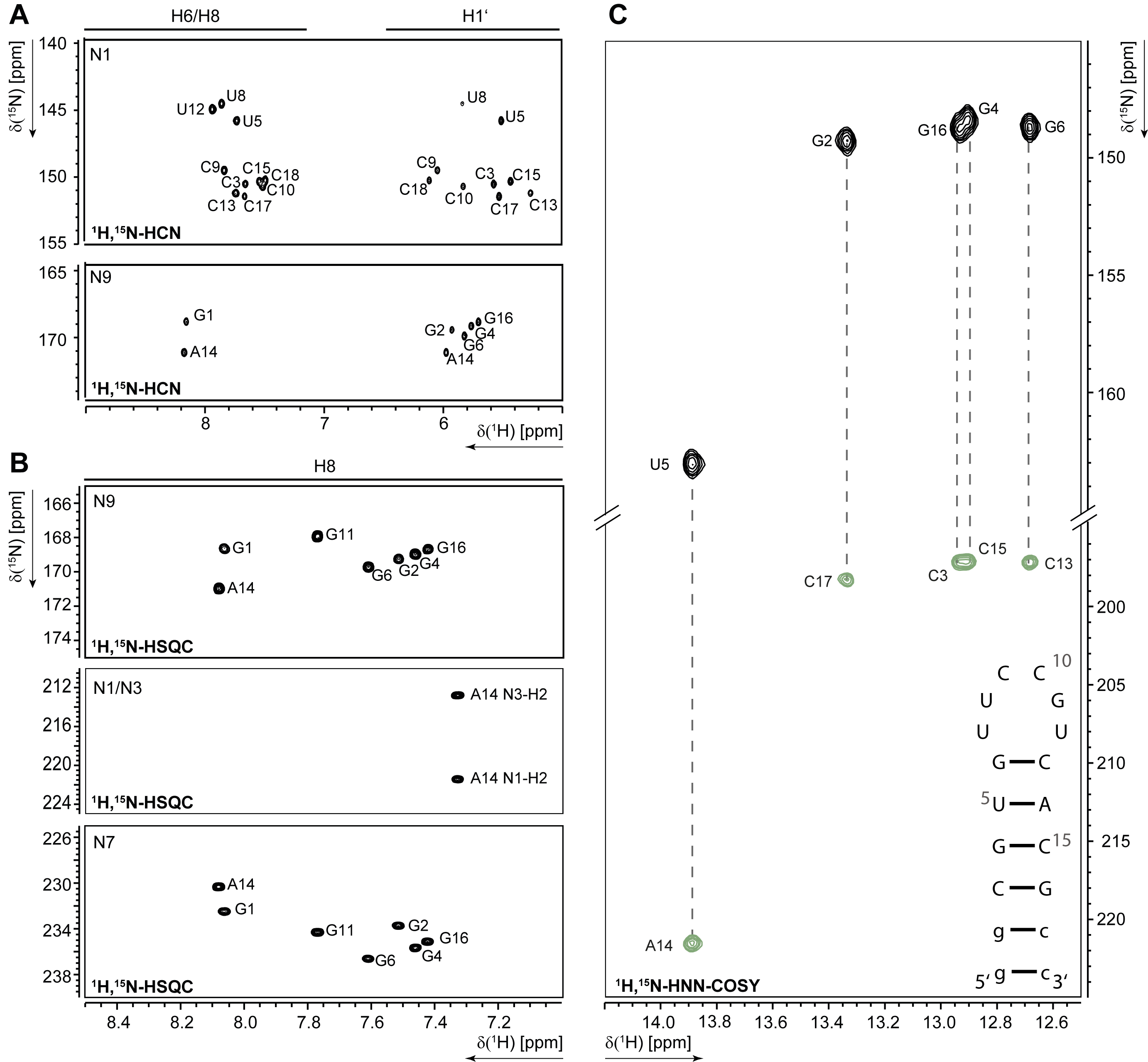


Figure SI 2: **A)** 2D plane of the ^1^H,^15^N-HCN experiment correlation the H6/H8 and H1’ resonances with the N1 and N9 resonances. **B)** ^1^H,^15^N-HSQC experiment showing the H8-N9/N7 correlations. **C)** ^1^H,^15^N-HNN-COSYand the secondary structure of the SL5a_WT RNA**.** All spectra were measured at 298 K with the uniformly ^13^C, ^15^N labelled 5SL5a_WT sample.


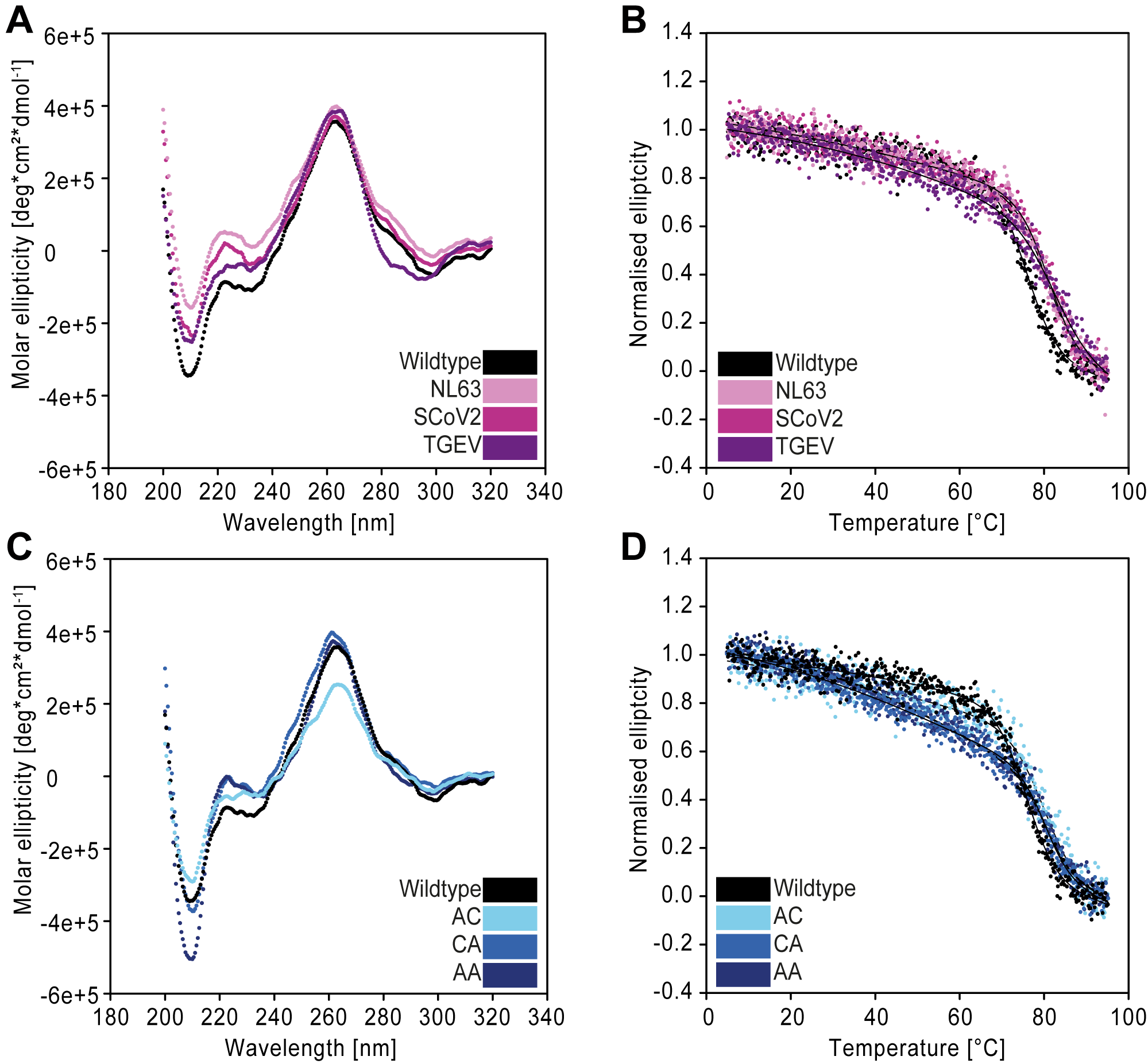


Figure SI 3: Circular dichroism-spectroscopic analysis of the different constructs, showing the variation in the stability due to different melting points. **A)** Circular dichroism spectra of the wildtype compared to the natural mutants. **B)** Thermal melting analysis of the wildtype compared to the natural mutants. **C)** Circular dichroism spectra of the wildtype compared to the artificial mutants. **D**) Thermal melting analysis of the wildtype compared to the artificial mutants. All samples had a concentration of 8 µM.
